# Intra-coronary morphine versus placebo in the treatment of acute ST-segment elevation myocardial infarction: the MIAMI randomized controlled trial

**DOI:** 10.1186/s12872-018-0936-8

**Published:** 2018-10-19

**Authors:** Philippe Le Corvoisier, Romain Gallet, Pierre-François Lesault, Etienne Audureau, Muriel Paul, Julien Ternacle, Saïd Ghostine, Stéphane Champagne, Raphaele Arrouasse, Dalila Bitari, Gauthier Mouillet, Jean-Luc Dubois-Randé, Alain Berdeaux, Bijan Ghaleh, Jean-François Deux, Emmanuel Teiger

**Affiliations:** 1 Department VERDI, Inserm, CIC1430, AP-HP, Henri Mondor Hospital, 51 Avenue du Maréchal de Lattre de Tassigny, F-94010 Creteil, France; 2 Inserm, U955 team 3, F-94010 Creteil, France; 3 Interventional Cardiology Unit, AP-HP, Henri Mondor Hospital, F-94010 Creteil, France; 4 Department of Public Health and CEPIA EA7376, AP-HP, Henri Mondor Hospital, F-94010 Creteil, France; 5 Department of Pharmacy, AP-HP, Henri Mondor Hospital, F-94010 Creteil, France; 6 Department of Cardiology, Marie-Lannelongue Hospital, F-92350 Le Plessis-Robinson, France; 7 Department of Cardiology, AP-HP, Henri Mondor Hospital, F-94010 Creteil, France; 8 Department of Radiology, AP-HP, Henri Mondor Hospital, F-94010 Creteil, France

**Keywords:** STEMI, Reperfusion injury, Cardioprotection, Infarct size, Morphine

## Abstract

**Background:**

Experimental studies suggest that morphine may protect the myocardium against ischemia-reperfusion injury by activating salvage kinase pathways. The objective of this two-center, randomized, double-blind, controlled trial was to assess potential cardioprotective effects of intra-coronary morphine in patients with ST-segment elevation myocardial infarction (STEMI) referred for primary percutaneous intervention.

**Methods:**

Ninety-one patients with STEMI were randomly assigned to intracoronary morphine (1 mg) or placebo at reperfusion of the culprit coronary artery. The primary endpoint was infarct size/left ventricular mass ratio assessed by magnetic resonance imaging on day 3–5. Secondary endpoints included the areas under the curve (AUC) for troponin T and creatine kinase over three days, left ventricular ejection fraction assessed by echocardiography on days 1 and 6, and clinical outcomes.

**Results:**

Infarct size/left ventricular mass ratio was not significantly reduced by intracoronary morphine compared to placebo (27.2% ± 15.0% vs. 30.5% ± 10.6%, respectively, *p* = 0.28). Troponin T and creatine kinase AUCs were similar in the two groups. Morphine did not improve left ventricular ejection fraction on day 1 (49.7 ± 10.3% vs. 49.3 ± 9.3% with placebo, *p* = 0.84) or day 6 (48.5 ± 10.2% vs. 49.0 ± 8.5% with placebo, *p* = 0.86). The number of major adverse cardiac events, including stent thrombosis, during the one-year follow-up was similar in the two groups.

**Conclusions:**

Intracoronary morphine at reperfusion did not significantly reduce infarct size or improve left ventricular systolic function in patients with STEMI. Presence of comorbidities in some patients may contribute to explain these results.

**Trial registration:**

ClinicalTrials.gov, NCT01186445 (date of registration: August 23, 2010).

## Background

Ischemic heart disease is the leading cause of morbidity and mortality worldwide. Although the prognosis of ST-segment elevation myocardial infarction (STEMI) has improved significantly in recent years [1], heart failure remains a common complication [2]. Early reperfusion of the infarct-related artery is the cornerstone of the modern treatment of STEMI. However, reperfusion of the ischemic myocardium can induce additional cell damage by triggering various abrupt biochemical changes [3, 4], including generation of reactive oxygen species, calcium overload, and opening of the mitochondrial permeability transition pore (mPTP) [4]. These mechanisms induce the death of cardiomyocytes that were viable at the end of the ischemic period, thereby exacerbating the myocardial injury [3]. Treatments to prevent or treat ischemia-reperfusion injury may therefore be of major clinical interest.

Morphine holds promise for preventing ischemia-reperfusion injury. Several experimental studies in rodents showed a reduction in infarct size after morphine injection at the time of reperfusion [5, 6]. The cardioprotective effects of morphine involve activation of the PI3-kinase pathway which enhances mitochondrial resistance to calcium overload and inhibits mPTP opening [6]. Other data suggest a role for nitric oxide and K_ATP_ channels [7, 8]. Similar cardioprotective effects have been reported for other opioid receptors agonists such as remifentanil [9].

The aim of this randomized, double-blind, placebo-controlled, two-center study in patients with STEMI was to evaluate whether intracoronary morphine administration at the time of reperfusion diminished myocardial injury assessed by magnetic resonance imaging (MRI).

## Methods

This randomized double-blind placebo-controlled trial compared intracoronary morphine to placebo in patients with STEMI.

### Study population

Eligible patients were men and women with STEMI referred for primary percutaneous coronary intervention (PCI) within 6 h after chest symptom onset. STEMI was defined as chest pain lasting more than 15 min with at least one of the following criteria: ST elevation ≥1 mm in at least two contiguous leads or presumed or certain appearance of a Q wave in three contiguous leads. Only patients with a TIMI coronary flow score of 0 on the first angiogram were included. Exclusion criteria were fibrinolysis, known allergy to morphine hydrochloride, uncontrolled epilepsy, brain injury or intracranial hypertension, previous STEMI or coronary artery bypass grafting (CABG), resuscitated cardiac arrest, cardiogenic shock or mechanical ventilation at admission, mechanical complication, sustained ventricular arrhythmia, high-grade atrioventricular block, decompensated respiratory insufficiency, serious hepatocellular insufficiency, and contraindication to cardiac MRI or gadolinium injection.

The study was performed in accordance with the ethical principles stated in the Declaration of Helsinki. The protocol was approved by the institutional review board of Pitie-Salpetriere hospital and all patients gave written informed consent. All data were measured off-line by a physician blinded to group allocation.

### Percutaneous coronary intervention

Patients in both the morphine and placebo groups routinely received intravenous aspirin and a loading dose of P2Y12 inhibitors before PCI. Intravenous heparin was administered to maintain an activated clotting time > 250 s.

All coronary angiography and stent implantation procedures were performed using standard interventional techniques. Thromboaspiration, balloon predilation, stent type selection, and use of glycoprotein IIb/IIIa inhibitors were at the operators’ discretion.

### Randomization procedure and study treatment administration

Randomization (1:1) sequences were generated by the biostatistician using random number tables and stratified by center using random blocks of 4. Pharmacists prepared sequentially numbered identical syringes of morphine (1 mg in 3 mL of saline) or placebo (3 mL of saline) according to the randomization sequence. Interventional cardiologists were blinded to allocation group. The study treatment was administered immediately after the artery was reopened (TIMI flow 2 or 3).

### MRI protocol

MRI was performed between day 3 and day 5 on a clinical 1.5 T system (Magnetom Avanto; Siemens Healthcare; Erlangen; Germany) using a 6-channel phased-array cardiac coil to assess myocardial injury. Unenhanced cine Steady State Free Precession (SSFP) sequences were acquired in the short axis plane. Eight to 10 sections encompassing the left ventricle were acquired for each patient. T2W–short tau inversion recovery (T2W-STIR) sequences were acquired in the short axis section covering the whole left ventricle using the following parameters (voxel size 2 × 1.5 × 8 mm, flip angle 90/180°, effective echo time 47 ms, bandwidth 235 Hz/pixel). Fifteen minutes after injection of 0.2 mmol/Kg of gadolinium (Dotarem; Guerbet; Aulnay sous Bois; France), late gadolinium enhanced (LGE) images were acquired using a segmented three-dimensional IR Gradient Echo T1-weighted technique. Left ventricular (LV) end-diastolic and end-systolic volume indexes and ejection fraction were measured from SSFP images using dedicated software (SyngoVia; Siemens Healthcare). The extent of myocardial edema was quantified on the T2W images using semiautomatic detection with the full width at half-maximum approach on a dedicated software (cvi42; Circle Cardiovascular Imaging Inc., Calgary, Alberta, Canada). Same approach (full width at half-maximum method) was used to calculate infarct size. LV mass was calculated by countering inner and outer boundaries of the LV wall on LGE images. Infarct size and LV mass were expressed in grams (myocardial density: 1.05 g/ml). The relative proportion of myocardial infarction was calculated by dividing infarct size by total LV mass.

### Transthoracic echocardiography

Transthoracic echocardiography was performed on days 1 and 6. Left ventricular ejection fraction (LVEF) was calculated from parasternal long axis and apical four-chamber views using the biplane Simpson’s method.

### Study endpoints

The primary endpoint was infarct size/LV mass ratio measured by MRI between days 3 and 5. Secondary endpoints included the ratio of infarct size over area at risk measured by MRI, the areas under the curves (AUCs) of serum creatine kinase and troponin T levels (measured every 4 h during the first 24 h then every 6 h during the next 48 h), LVEF measured by echocardiography on days 1 and 6, infarct size/LV mass ratio by MRI after 1 year, and clinical outcome after 3 months and 1 year.

### Sample size estimation

A previous study reported a mean infarct size measured by MRI of 14% ± 6% of LV mass [10]. The estimated relative decrease in infarct size/LV mass after intracoronary morphine in preclinical studies was at least 33% [6]. To have 90% power for detecting a 33% decrease with alpha set at 5%, 36 patients were needed in each group. Assuming that 30% of patients would not be evaluable (e.g., due to a contraindication to MRI, consent withdrawal, or poor MRI acquisition), 91 patients were included in this study.

### Statistical analysis

Continuous variables are described as mean ± standard deviation and categorical variables as numbers (%). For the primary outcome, patients were assessed according to randomized treatment group under the intent-to-treat (ITT) principle. Continuous variables were compared using the Student or Mann-Whitney test, depending on the normality of distributions as assessed by the Shapiro-Wilk test, and categorical variables using the chi-square test or Fisher’s exact test, as appropriate. Pearson or Spearman correlation coefficients were computed to assess relationships between selected continuous parameters (i.e. troponin T and creatine kinase AUCs against infarct size/left ventricular mass ratio), as appropriate. All *p* values were two-sided, and *p* values lower than 0.05 were considered statistically significant. All analyses were performed using Stata V14.1 (StataCorp, College Station, TX, USA).

## Results

### Population characteristics

**Fig. 1:**
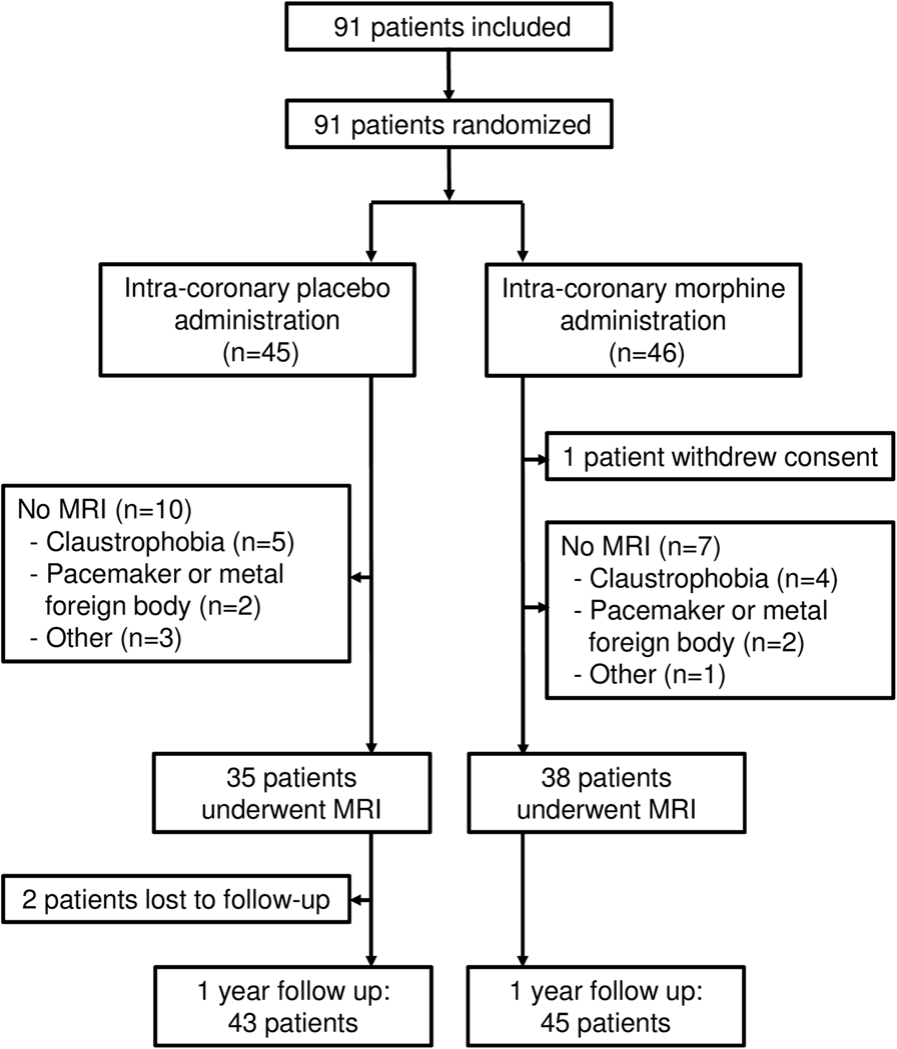
Patient flowchart

We included 91 patients with STEMI, who were randomly assigned to intracoronary injection of either morphine or placebo at the time of reperfusion. One patient withdrew consent after study inclusion, leaving 90 patients in the intention-to-treat population (Fig. 1).

There was no difference in baseline characteristics and concomitant therapies between both groups. The distribution of the culprit arteries was well balanced between the two arms. Most patients (93.3%) received glycoprotein (GP) IIb/IIIa receptor inhibitors or bilavirudin (Table 1).

**Table 1:** Patient characteristics

	Morphine group (*n* = 45)	Placebo group (*n* = 45)
Patient characteristics		
Age, y^a^	59.9 ± 13.2	56.5 ± 10.6
Male/female, n (%)	36 (80.0)/9 (20.0)	37 (82.2)/8 (17.8)
Diabetes mellitus, n (%)	8 (17.8)	5 (11.1)
Hypercholesterolemia, n (%)	14 (31.1)	13 (28.9)
Smokers (current or former), n (%)	33 (73.3)	31 (68.8)
Hypertension, n (%)	20 (44.4)	16 (35.6)
Body mass index, kg/m^2 a^	27.0 ± 4.1	26.0 ± 2.9
Medical history, n (%)		
History of coronary artery disease	4 (8.9)	4 (8.9)
History of heart failure	0 (0.0)	0 (0.0)
Clinical characteristics		
Symptom-to-balloon time, min^a^	189 ± 87	193 ± 108
Killip class > 1, n (%)	1 (2.2)	1 (2.2)
Sustained ventricular arrhythmia before admission, n (%)	0 (0.0)	2 (4.4)
Location of the culprit lesion, n (%)		
Left anterior descending artery	23 (51.1)	20 (44.4)
Circumflex coronary artery	4 (8.9)	8 (17.8)
Right coronary artery	18 (40.0)	17 (37.8)
Rentrop score, n (%)		
0	28 (62.2)	29 (64.5)
1	10 (22.2)	10 (22.2)
2–3	7 (15.6)	6 (13.3)
Number of diseased vessels, n (%)		
1	34 (75.6)	36 (80.0)
≥ 2	11 (24.4)	9 (20.0)
Concomitant therapy, n (%)		
Aspirin	45 (100.0)	44 (97.8)
P2Y12 receptor blockers	44 (97.8)	44 (97.8)
GPIIb-IIIa receptor inhibitors/bilavirudin	22(48.9)/20(44.4)	26(57.7)/16(35.6)
Intravenous morphine injection	11 (24.4)	11 (24.4)

^a^Mean ± SD

### Safety of intracoronary injection

Intracoronary injection of the study medication was associated with asymptomatic hypotension in two patients, one in each group. At the end of the procedure, the groups had no significant differences in rates of slow flow, distal embolism, or intra-stent residual thrombosis. The proportions of patients with myocardial blush grade II (21.1% vs. 30.0% in morphine and placebo groups, respectively) or III (47.4% vs. 40.0%) were similar in the two groups (*p* = 0.96) (Table 2).

**Table 2:** Safety of intracoronary injection

	Morphine group (*n* = 45)	Placebo group (*n* = 45)	*P* value
Safety of IC injection, n (%)			
Hypotension	1 (2.2)	1 (2.2)	1.00
Ventricular arrhythmia	0 (0.0)	0 (0.0)	1.00
PCI results, n (%)			
Slow flow	1 (2.2)	1 (2.2)	1.00
Intra-stent residual thrombosis	3 (6.7)	7 (15.6)	0.18
Distal embolism	2 (4.4)	3 (6.7)	1.00

*IC* intracoronary, *PCI* percutaneous intracoronary intervention

### Infarct size

Myocardial injury was assessed by MRI (Fig. 1). Infarct size/LV mass ratio was not significantly improved by intracoronary morphine (27.2 ± 15.0% vs. 30.5 ± 10.6% with placebo, *p* = 0.28). Infarct size remained similar in the two groups when adjusted on the area at risk (Table 3). A per-protocol analysis led to the same conclusion as did the intention-to-treat analysis.

**Table 3:** Myocardial injury assessed by MRI

	Morphine group (*n* = 38)	Placebo group (*n* = 35)	*P* value
LV mass, g	131 ± 34	128 ± 33	0.63
Infarct size, g	36.6 ± 22.5	39.5 ± 17.7	0.54
Infarct size/LV mass, %	27.2 ± 15.0	30.5 ± 10.6	0.28
Area at risk/LV mass, %	31.3 ± 12.5	36.0 ± 11.2	0.15
Infarct size/Area at risk, %	80.5 ± 19.4	84.1 ± 19.5	0.49
LV ejection fraction, %	49.2 ± 11.0	47.6 ± 8.7	0.50
LV end-diastolic volume, mL/m^2^	82.2 ± 16.8	78.4 ± 21.6	0.41
LV end-systolic volume, mL/m^2^	42.5 ± 14.9	41.7 ± 15.7	0.83

*LV* left ventricle

**Fig. 2:**
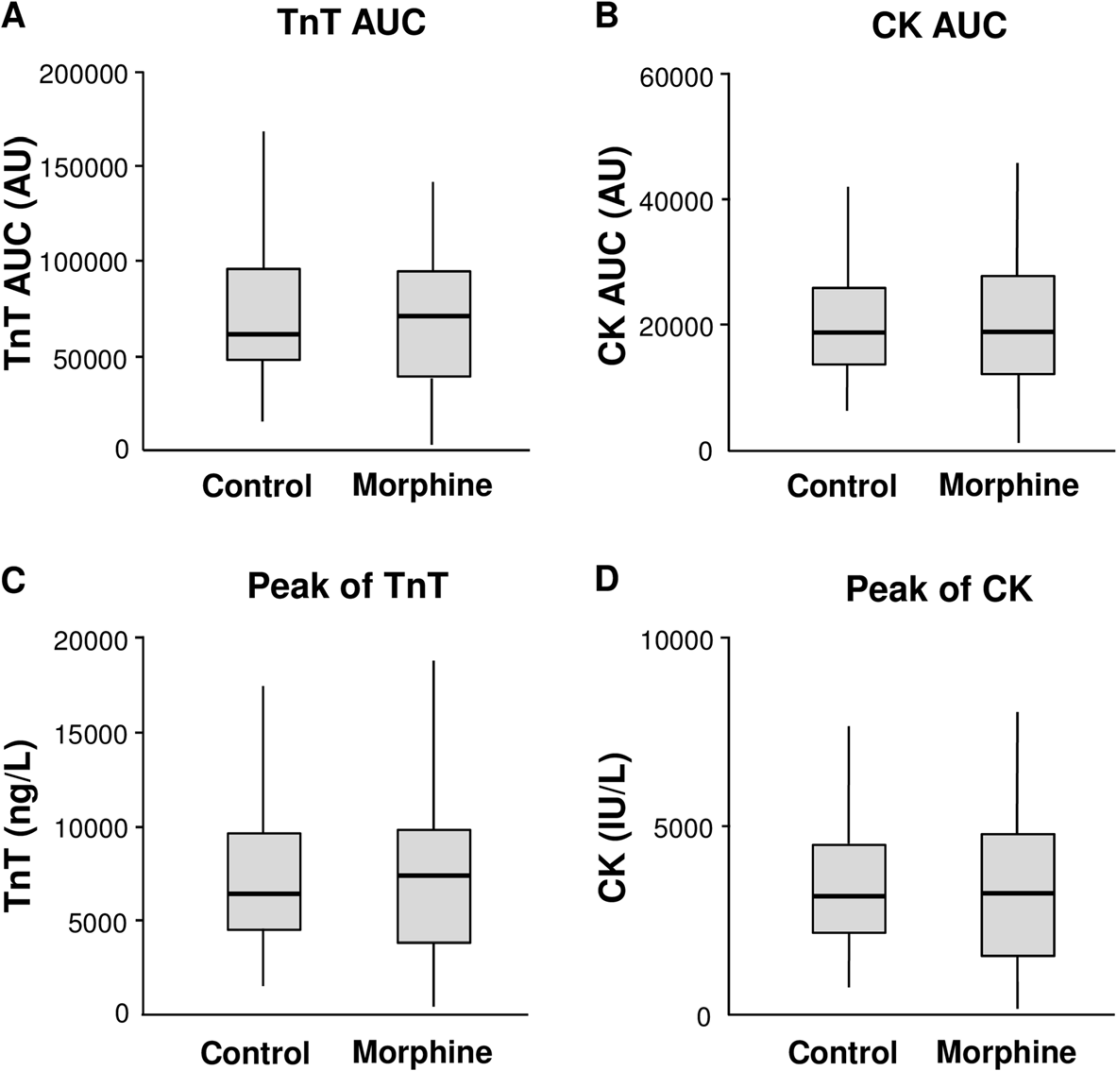
Areas under the curves and peak of serum troponin T (TnT) (Panel **a** and **c**) and creatine kinase *CK* (panel **b** and **d**) levels. No difference was observed between groups. Results are shown as boxplots, with each box representing the interquartile range (1st to 3rd quartile, IQR), the line within the box indicating the median, and the whiskers extending to 1.5 times the IQR above and below the box

These results are supported by cardiac enzyme release. Morphine did not significantly reduce AUC_0–72 h_ values for troponin T (70,979 [36,726-100,070] A.U. vs. 61,443 [46,330-97,460] A.U. with placebo, *p* = 0.71) and creatine kinase (18,815 [11,829-28,389] A.U. vs. 18,721 [13,515-25,994] A.U. with placebo, *p* = 0.79). Neither were peak troponin T or creatine kinase values significantly different (Fig. 2). Infarct size assessed by MRI correlated strongly with enzyme release (troponin T: *r* = 0.73, *p* < 0.0001; creatine kinase: *r* = 0.68, *p* < 0.0001).

### Left ventricular systolic function

LVEF assessed by echocardiography was similar in the morphine and placebo groups on day 1 (49.7% ± 10.3% vs. 49.3% ± 9.3%, *p* = 0.84) and day 6 (48.5% ± 10.2% vs. 49.0% ± 8.5%, *p* = 0.86). The percentage of patients with LV systolic dysfunction defined as LVEF below 45% was not significantly lower with morphine (29.5% vs 26.7% with placebo, *p* = 0.76). Diastolic function assessed based on the E/E’ ratio was similar in the two groups (8.2 ± 2.5 vs 7.9 ± 3.5, *p* = 0.73). Similarly, MRI showed no significant differences in LV structural or functional parameters on day 3–5 (Table 3).

### One-year outcomes

MRI performed after one year showed similar infarct size/LV mass ratio (21.4 ± 9.8% vs. 23.0 ± 8.0% in placebo, *p* = 0.51) and LVEF (52.2% ± 10.3% vs. 53.3% ± 11.4%, *p* = 0.71) values in the two groups. The frequency of major adverse cardiac events (MACEs) after 12 months was not lower in the morphine group: MACEs occurred in 7 (15.6%) patients in the morphine group and 8 (18.6%) patients in the placebo group. The most common MACE was unplanned admission for decompensated heart failure. Stent thrombosis occurred in one patient in each group. All MACEs were ascribed by the investigators to the underlying cardiovascular disease (Table 4).

**Table 4:** One-year clinical outcomes

	Morphine group (*n* = 45)	Placebo group (*n* = 43)	*P* value
Major adverse cardiac events, n (%)	7 (15.6)	8 (18.6)	0.70
Cardiovascular death	0 (0.0)	1 (2.3)	
Heart failure	2 (4.4)	2 (4.6)	
STEMI	1 (2.2)	1 (2.3)	
Stable angina	3 (6.7)	3 (7.0)	
Sustained ventricular arrhythmia	1 (2.2)	1 (2.3)	
Other clinical events, n (%)	5 (11.1)	4 (9.3)	0.78
Non-cardiovascular death	1 (2.2)	0 (0.0)	
CABG	1 (2.2)	2 (4.6)	
Elective PCI	3 (6.7)	2 (4.6)	

*CABG* coronary artery bypass grafting, *PCI* percutaneous coronary intervention, *STEMI* ST-elevation myocardial infarction

### Subgroup analyses

Post-hoc analyses were performed to examine the consistency of results across subgroups and to generate hypotheses to explain the study results. All tests for interaction were non-significant for age, sex, symptom-to-balloon time, and hypercholesterolemia, with *p* values > 0.20. Diabetes was common in the study population and may have been a confounder. The test for interaction between diabetes and treatment effect found a *p* value of 0.17. An analysis in the subgroup without diabetes showed a trend toward an improvement in the primary endpoint in the morphine group (22.5% [14–33] vs. 27.5% [22–41], *p* = 0.053). In contrast, no effect was evidenced in the subgroup of diabetic patients (35% [29–48] vs. 31% [28–36], *p* = 0.36).

## Discussion

The main finding from this randomized controlled trial was that intracoronary morphine, when added to an optimal reperfusion strategy, did not significantly alleviate reperfusion injury or improve secondary endpoints in patients with STEMI. Intracoronary morphine was safe, and serious adverse events occurred with similar frequencies in the two groups.

In recent years, many experimental studies suggested that opioid receptor agonists used in everyday practice might protect the heart against ischemia-reperfusion injury [5, 6]. A few clinical trials evaluated whether these preclinical data translated into clinical benefits, in various settings. A small pilot study showed a reduction in myocardial ischemia after intracoronary morphine during elective PCI [11]. In another randomized controlled trial in patients with STEMI, intravenous morphine injection combined with remote ischemic postconditioning improved the resolution of ST-segment elevation [12]. In contrast, intracoronary morphine failed to reduce myocardial injury assessed by MRI in an open-label randomized study in patients with STEMI, among whom less than 10% received GP IIb/IIIa receptor inhibitors [13].

The potential cardioprotective effect of opioid agonists has also been investigated during cardiac surgery. Murphy et al. reported that morphine administration improved LV function recovery after CABG compared to fentanyl [14]. A possible explanation is the greater affinity of morphine for δ-opioid receptor subtypes. Similarly, morphine-induced postconditioning reduced cardiac injury in patient undergoing tetralogy of Fallot correction [15]. Another randomized controlled trial demonstrated a cardioprotective effect of the opioid agonist remifentanil during CABG in patients managed with standardized anesthesia protocol [16]. The need for inotropic support after the intervention was reduced in patients given remifentanil. Taken together, these studies suggest that opioid agonists may have a cardioprotective effect during cardiac surgery.

The MIAMI study was designed to assess the potential cardioprotective effects of morphine in STEMI patients. The dose was selected based on previous experimental studies [6]. Intracoronary injection was chosen to allow a rapid activation of target receptors without systemic effect. However, in our study, intracoronary morphine did not significantly reduce reperfusion injury in patients with STEMI undergoing optimal reperfusion therapy. Most of the clinical studies designed to translate experimental findings on cardioprotection to clinical settings produced mixed or negative results [17, 18]. Several hypotheses may explain these discrepancies. Some comorbidities may jeopardize the effectiveness of cardioprotective strategies after myocardial ischemia-reperfusion [19]. In experimental models, the effect of ischemic postconditioning is reduced or even abolished in animal models with hypercholesterolemia or diabetes [20, 21]. Loss of cardioprotection was associated with impaired activation of the reperfusion injury salvage kinase pathway [7, 22, 23]. No clinical trial has specifically investigated the effect of pharmacological cardioprotection in patients with comorbidities. However, Yetgin et al. showed in 634 patients treated with multiple balloon inflations during primary PCI, that the effect of ischemic postconditioning was more pronounced in the absence of diabetes [24]. Therefore, we cannot exclude that the presence of comorbidities in some patients contributed to explain our results.

Recently, concern has been raised about the use of intravenous morphine as an analgesic in patients with STEMI [25]. Several studies demonstrated interactions between morphine and P2Y12 receptor inhibitors, leading to impaired gastrointestinal absorption, decreased concentration of active metabolites, and delayed antiplatelet activity [26, 27]. Several strategies have been proposed to overcome this interaction, including concomitant administration of GP IIb/IIIa receptor inhibitors [25, 26]. The most clinically relevant issue is whether alterations in the pharmacokinetics of P2Y12 receptor inhibitors affect clinical outcomes in patients simultaneously treated with multiple antithrombotic drugs. A subgroup analysis of data from the ATLANTIC study showed that morphine therapy may interact with ST-segment elevation resolution before PCI in patients with STEMI [28]. However, this finding was not associated with a difference in infarct-related artery patency at the time of primary PCI. The impact of preadmission intravenous morphine therapy on patient outcomes was assessed using data from the prospective multicenter nationwide FAST-MI registry [29]. Of the 2438 patients with STEMI, 19% received morphine. After adjustment for baseline characteristics, morphine therapy did not predict the one-year clinical outcome. Results were similar in the subgroup of patients treated with P2Y12 receptor inhibitors before hospital admission [29]. Similarly, in another cohort of 969 patients with acute anterior STEMI, prehospital morphine therapy was not associated with one-year mortality [30].

Both the route of administration and the dose of morphine differed markedly between these previous studies and the present study. However, our trial adds new evidence to the morphine debate. Intracoronary morphine had no adverse effect on infarct size in randomized patients. Clinical outcomes were similar with morphine and the placebo. The overall rate of serious adverse events was comparable to that seen in a recent similar trial.

One limitation of this study should be noted. Our study was powered to detect a difference in the primary endpoint, and the subgroup analyses remain exploratory.

## Conclusions

The MIAMI trial reported here assessed whether adding intracoronary morphine to optimal PCI diminished reperfusion injury in patients with STEMI. Intracoronary morphine was safe but did not significantly diminish LV infarct size or improve LV systolic function.

## Abbreviations

AUC: Area under the curve
CABG: Coronary artery bypass grafting
GP IIb/IIIa receptor inhibitors: Glycoprotein IIb/IIIa receptor inhibitors
LVEF: Left ventricular ejection fraction
mPTP: Mitochondrial permeability transition pore
MRI: Magnetic resonance imaging
PCI: Percutaneous coronary intervention
STEMI: ST-segment elevation myocardial infarction
